# Preclinical study of CD19 detection methods post tafasitamab treatment

**DOI:** 10.3389/fimmu.2023.1274556

**Published:** 2023-10-20

**Authors:** Kristina Ilieva, Markus Eberl, Jan Jaehrling, Derek Blair, Maria Patra-Kneuer, Rainer Boxhammer, Diana Alvarez Arias, Christina Heitmüller

**Affiliations:** ^1^ Department of Translational Research, Translational Sciences, MorphoSys AG, Planegg, Germany; ^2^ Department of Analytical Sciences, Translational Sciences, MorphoSys AG, Planegg, Germany; ^3^ Department of Clinical Biomarkers & Companion Diagnostics, Translational Sciences, MorphoSys AG, Planegg, Germany; ^4^ Translational Sciences, Incyte Corporation, Wilmington, DE, United States

**Keywords:** antibody immunotherapy, DLBCL, tafasitamab, CD19 detection, flow cytometry, immunohistochemistry, antibody competition, antigen masking

## Abstract

**Introduction:**

Several CD19 targeted antibody-based therapeutics are currently available for patients with diffuse large B-cell lymphoma (DLBCL), including the Fc-modified antibody immunotherapy tafasitamab. This therapeutic landscape warrants the evaluation of potential sequencing approaches. Prior to a subsequent CD19-targeted therapy, CD19 expression on tafasitamab-treated patient biopsy samples may be assessed. However, no standardized methods for its detection are currently available. In this context, selecting a tafasitamab-competing CD19 detection antibody for immunohistochemistry (IHC) or flow cytometry (FC) may lead to misinterpreting epitope masking by tafasitamab as antigen loss or downregulation.

**Methods:**

We analyzed a comprehensive panel of commercially available CD19 detection antibody clones for IHC and FC using competition assays on tafasitamab pre-treated cell lines. To remove bound tafasitamab from the cell surface, an acidic dissociation protocol was used. Antibody affinities for CD19 were measured using Surface Plasmon Resonance (SPR) or Bio-Layer Interferometry (BLI).

**Results:**

While CD19 was successfully detected on tafasitamab pre-treated samples using all 7 tested IHC antibody clones, all 8 tested FC antibody clones were confirmed to compete with tafasitamab. An acidic dissociation was demonstrated essential to circumvent CD19 masking by tafasitamab and avoid false negative FC results.

**Discussion:**

The current study highlights the importance of selecting appropriate CD19 detection tools and techniques for correct interpretation of CD19 expression. The findings presented herein can serve as a guideline to investigators and may help navigate treatment strategies in the clinical setting.

## Introduction

A range of CD19 targeted therapies have been approved for the treatment of patients with DLBCL. In 2020, the anti-CD19 Fc-modified antibody immunotherapy tafasitamab received accelerated approval for patients with relapsed or refractory (r/r) DLBCL, not eligible for autologous stem cell transplant, in combination with the immunomodulatory drug lenalidomide (1). Tafasitamab, in combination with lenalidomide and R-CHOP, is currently being tested as a frontline therapy for newly diagnosed DLBCL patients (NCT04824092, frontMIND). Other anti-CD19-targeted DLBCL therapies include the antibody-drug conjugate loncastuximab tesirine, currently approved for r/r DLBCL patients after at least two previous lines of therapy, and the anti-CD19 chimeric antigen receptor T-cell (CART19) therapies axicabtagene-ciloleucel (axi-cel) and lisocabtagene maraleucel (liso-cel), approved as a second line of therapy and tisagenlecleucel (tisa-cel), as a third line of therapy (2–5).

The availability of different anti-CD19 therapies opens possibilities for therapeutic sequencing. Reportedly, CART19 treatment may induce CD19 loss, highlighting the importance of CD19 expression monitoring post-treatment (6, 7). To confirm target expression prior to a subsequent anti-CD19 therapy, biopsy samples from patients treated with tafasitamab may be analyzed using flow cytometry (FC) or immunohistochemistry (IHC). At present, CD19 detection methods in routine clinical practise are not universal, with different institutions utilising different platforms and different commercially available CD19 detection antibodies. The target epitopes of most commercially available CD19 detection antibodies are often unknown to end users for business reasons, and it is unclear whether they compete with tafasitamab. Fine epitope mapping of the CD19 extracellular domain has demonstrated that three commonly used anti-CD19 antibody clones (FMC63, 4G7-2E3, and 3B10) bind overlapping epitopes of CD19 (8), and tafasitamab is derived from the clone 4G7 (9). Importantly, using a tafasitamab-competing antibody to detect CD19 on tafasitamab-treated samples may lead to signal reduction and confusion of CD19 epitope masking with antigen loss.

The current study aims to evaluate a comprehensive set of commercially available anti-CD19 antibodies on tafasitamab pre-treated cell lines by FC and IHC.

## Method

### Cell lines

Raji (Burkitt lymphoma (BL)), MEC-1 (B-cell chronic lymphocytic leukemia (B-CLL)), JVM-2 (B-cell prolymphocytic leukemia (B-PLL)), U-2932 (Diffuse Large Cell B-cell Lymphoma (DLBCL)), SU-DHL-4 (DLBCL), and SU-DHL-6 (DLBCL) cell lines were purchased from DSMZ (Deutsche Sammlung von Mikroorgnismen und Zellkulturen, Cat# ACC 319, ACC 497, ACC 12, ACC 633, ACC 495 and ACC 572) and SU-DHL-2 (DLBCL) was purchased from ATCC (American Tissue Culture Collection, Cat# CRL-2956). Raji, JVM-2, U-2932 and SU-DHL-2 cells were cultured in RPMI (Roswell Park Memorial Institute) medium supplemented with 10% fetal calf serum (FCS) and 1% GlutaMax, SU-DHL-4 and SU-DHL-6 in RPMI with 20% FCS and 1% Glutamax while MEC-1 cells were cultured in IMDM (Iscove´s Modified Dulbecco´s Medium) with 10% FCS. All cells were incubated in a humidified atmosphere, at 5% CO_2_. CD19 expression on the cells was quantified using the BD Quantibrite™ system, according to the manufacturer´s instructions and PE-labeled anti-CD19 (Biolegend, clone HIB19) as described previously (10).

### Antibodies

Tafasitamab was provided by MorphoSys AG. Tafasitamab-AF488 was conjugated using AlexaFluor™ 488 C_5_ maleimide. The amino acid sequences of the variable regions of RB4 (loncastuximab) were obtained from the Inxight Drugs database of the National Center for Advancing Translational Sciences (NCATS). RB4 was recombinantly produced in house, as human IgG1-kappa. In addition, RB4 was expressed in house bearing a fluorescent mScarlet tag.

### Immunohistochemistry

Raji, MEC-1 and JVM-2 were either left untreated or incubated with 50 nM tafasitamab for 30 min at +4°C and washed thrice. 5E+07 cells per condition were fixed overnight in 10% Formalin. Before paraffin embedding, fixed cells were encapsulated into 200µL Histo Gel (Epredia, Cat# HG-4000-012) to form pellets. After paraffin embedding, 4µm thick sections were used for staining and heat induced antigen retrieval was performed at 95°C for 35min using either 10mM citrate buffer pH 6.0 (Sigma Aldrich, Cat# C9999-1000ML) + 0.05% Tween-20 (Merck, Cat# 8.22184.0500) or 1mM EDTA buffer pH 8.5 (Sigma Aldrich, Cat# E1161-1000ML) +0.05% Tween-20.

Following antigen retrieval, slides were treated with 3% fresh hydrogen-peroxide (Carl Roth, Cat# 1A8Y.3) for 10min at room temperature (RT) and blocking was performed for 60min at RT using 5% goat serum (Jackson, Cat# 005-000-121) in PBS. Primary antibodies were diluted in Dako antibody diluent (Dako, Cat# S0809), while detection antibodies were diluted in Dulbecco´s phosphate buffered saline (D-PBS). For additional information see antibody **Tables S1** and **S2**.

DAB Enhanced Liquid Substrate System (Sigma, Cat# D3939-1SET) was used for signal detection and no hematoxylin counterstaining was performed before slide mounting. Staining quantification was performed using ImageJ Software (NIH, version 1.53f51) and GraphPad Prism (version 8.4.3.).

### Flow cytometry competition assays

All antibodies were diluted in FACS buffer consisting of D-PBS supplemented with 3% FCS. Raji, MEC-1 or JVM-2 cells (5E+04 per test) were incubated with different concentrations of tafasitamab (ranging from 50 to 0.00064 nM, 5-fold titration) for 20 min on ice. Cells were washed with FACS buffer 3 times and incubated with a saturating concentration (50 nM) of a PE-labelled CD19 detection antibody for 20 min on ice, protected from light (**Table S3**, **Figure S7**). In case the concentration of the commercial CD19 detection antibody was unknown (LT19 and REA675, **Table S3**) or too low (J3-119, 4 µg/mL, **Table S3**), antibodies were used at the supplier recommended concentration per test. Antibody clone HD37 was used unconjugated and detected using a secondary anti-rabbit antibody (**Table S4**). Next, cells were washed 3 times using FACS buffer and analyzed using FACS Verse I. DAPI (4’,6-diamidino-2-phenylindole) was used as a live/dead differentiator. Data analysis was performed using FlowJo software v10.5.

### Acidic dissociation assay

U-2932, SU-DHL-1, -4 and -6 cells were incubated with different concentrations of tafasitamab (ranging from 200 to 0.2 µg/mL, 10-fold titration) and washed, as described above. Next, cells were either left untreated or treated with an acidic dissociation buffer to remove pre-bound tafasitamab off the cell surface. Cells were plated in 96 KingFisher Deepwell V-bottom plates (Thermo Scientific, #95040455) and re-suspended via pipetting (a minimum of 10 times) in 200 µL acidic dissociation buffer (D-PBS + 3% FCS, pH 2.1 adjusted with HCl). The suspension was neutralized using 1200 µL ice-cold FACS buffer (a minimum of 5 times re-suspension via pipetting) and spun down. The procedure was repeated three times before staining using the tafasitamab-competing PE-conjugated anti-CD19 antibody clone (HIB19). The samples were analyzed as described above.

## Results

**Figure 1:**
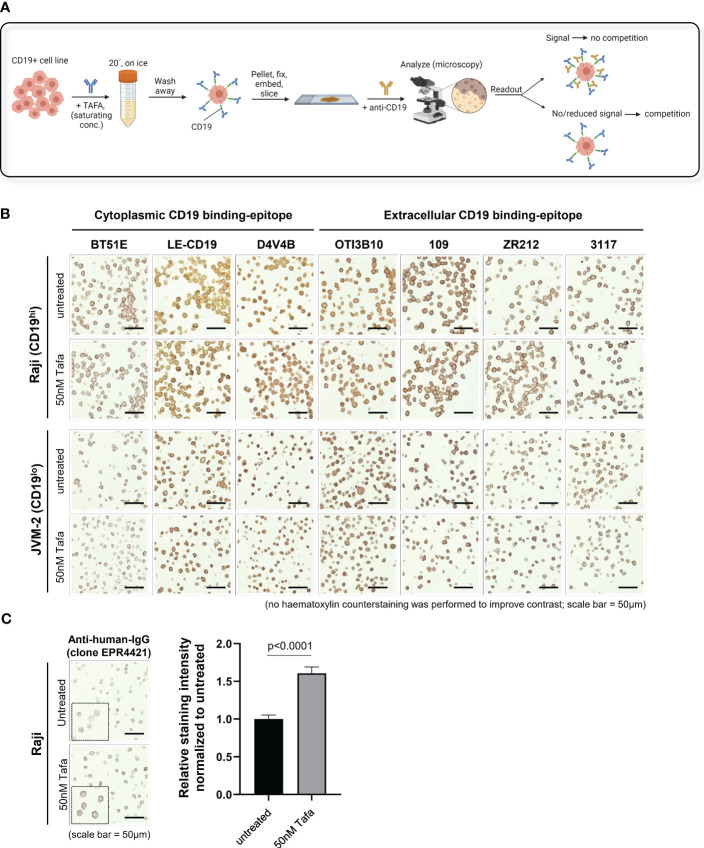
CD19 can be detected by IHC using commercial antibodies independent of tafasitamab pretreatment. **(A)** Schematic of an IHC workflow designed to assess TAFA competition with commercial anti-CD19 antibody clones for IHC. CD19+ cell lines were pre-incubated with saturating concentrations of TAFA (50 nM), washed, and processed to form a cell pellet. The pellets were formalin-fixed, paraffin-embedded (FFPE), sectioned, and stained with commercial anti-CD19 antibodies. Competition was confirmed when a reduction in signal was observed on TAFA pre-treated samples. Schematic was created using BioRender.com. **(B)** IHC competition experiments. Raji and JVM-2 cells were processed as described above. CD19 detection antibodies against the cytoplasmic tail of CD19 (clones BT51E, LE-CD19, D4V4B) and the extracellular domain of CD19 (clones OTI3B10, 109, ZR212, 3117) were tested. Samples were analyzed using the following equipment/parameters: Axiolab 5 microscope, Zeiss Axiocam 208 color camera, Zeiss N-Achroplan objective, magnification = 40x, numerical aperture = 0.65, at room temperature. Images were processed using the Zeiss Axiocam 208 color camera built-in software (firmware version 1.3.6) **(C)** To confirm that TAFA is still present in the FFPE samples and not lost during cell pellet processing, untreated and TAFA pre-treated samples were stained with anti-human IgG. Sample acquisition and analysis specifics were as described above. Quantification was performed with 30 cells per condition and relative staining intensity normalized to untreated cells is shown. Statistical analysis: Mann-Whitney test.

Using an antibody competition approach, we evaluated 7 anti-CD19 antibodies for IHC and 8 for FC (**Tables S1**, **S3**). The IHC assays were performed on formalin-fixed paraffin-embedded (FFPE) cell pellets, generated using the high and low CD19 expressing cell lines Raji and JVM-2, respectively. Prior to fixation, the cells were incubated with tafasitamab at a saturating concentration (50 nM) or left untreated (**Figure 1A**). CD19 was confirmed completely blocked by tafasitamab (**Figure S2**). Before proceeding with competition experiments using the cell pellets, optimal staining conditions were established using FFPE human tonsil samples. We tested 12 commercially available CD19 detection antibodies for IHC and successfully established staining protocols for 7 clones: 3 targeting the intracellular and 4 the extracellular domain of CD19 (**Table S1**). Next, the established clones were tested on tafasitamab-treated Raji (CD19^hi^) and JVM-2 (CD19^low^) cell pellet samples, alongside untreated controls to assess antibody binding competition (**Figures 1A**, **S1**). Thus, reduction or absence of a signal on tafasitamab-treated samples, compared to untreated controls, would indicate CD19 masking by tafasitamab (**Figure 1B**). No differences in CD19 surface staining pattern were observed on tafasitamab-treated vs. untreated samples with any of the 7 antibody clones tested. This result was in line with expectations for all antibodies targeting the intracellular domain of CD19 (BT51E, LE-CD19 and D4V4E). To confirm that tafasitamab was indeed still present in the FFPE samples and not lost during FFPE processing, tafasitamab was detected using two different anti-human IgG antibodies, as demonstrated in **Figures 1C**, **S4**. Prior to this experiment, low/lack of surface IgG expression on Raji and JVM-2 was also confirmed using FC, thus excluding potential interference with tafasitamab detection (**Figure S3**). Furthermore, our experiments highlighted another important feature of the IHC technique, to be kept in consideration: the method does not allow for quantitative evaluation of differential antigen expression. Despite the ~5-fold difference in CD19 expression between Raji and JVM-2 cells, quantified by FC, IHC could not discriminate between these levels and staining intensity can easily be affected by duration of chromogen incubation (**Figures 1B**, **S1**, **S5A**).

**Figure 2:**
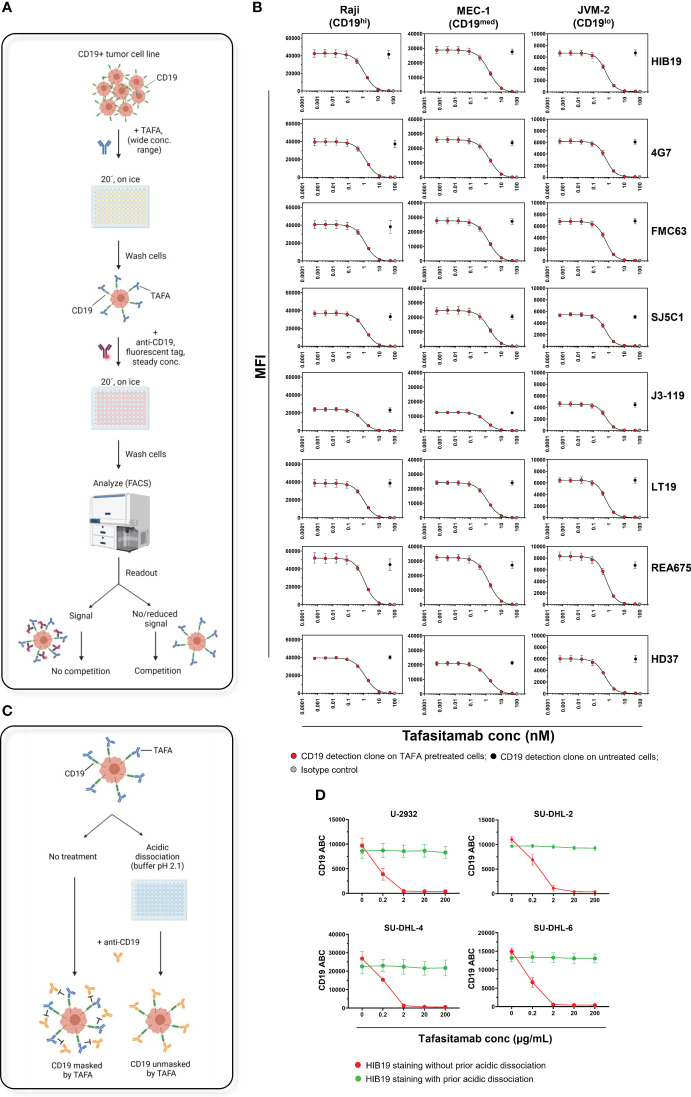
CD19 masking by tafasitamab does not allow for CD19 detection by FC using commercial monoclonal antibodies. **(A)** Schematic of a FC workflow designed to assess TAFA competition with commercially available anti-CD19 antibody clones. CD19+ tumor cell lines were pre-incubated with different concentrations of TAFA for 20 min on ice, washed, and incubated with a fluorescently labelled commercially available anti-CD19 antibody. Competition between the two antibodies was confirmed by measuring reduction/loss of fluorescent signal from the commercial clone using FC. Schematic was created using BioRender.com. **(B)** Flow cytometry competition experiments. Raji (Burkitt lymphoma, ~105000 CD19 ABC), MEC-1 (B-CLL, ~66000 CD19 ABC) and JVM-2 (B-PLL, ~20000 CD19 ABC) cells were processed as described above. Commercially available clones HIB19, 4G7, FMC63, SJ24C1, J3-119, LT19, REA675 and HD37 were tested. N≥3 individual experiments. **(C)** Schematic of an acidic dissociation procedure, which allows removal of pre-bound TAFA and exposure of CD19 on cell line samples for binding to a competing anti-CD19 antibody applied e.g. for detection of CD19 surface levels (right). Cell lines, pre-incubated with TAFA underwent 3 rounds of dissociation using a low pH buffer (pH=2.1). Without the acidic dissociation step, CD19 would be masked by TAFA and not accessible to a competing anti-CD19 antibody (left). **(D)** Four DLBCL cell lines (U-2932, SU-DHL-2, -4 and -6) were incubated for 20 min on ice with different concentrations of TAFA. After a wash, cells were incubated with the competing clone HIB19 at a concentration of 50 nM, with and without prior acidic dissociation. The samples were analyzed using flow cytometry. Detectable CD19 molecules were quantified using the Quantibrite system, BD. N=3 individual experiments. *B-CLL, B-cell Chronic Lymphocytic Leukemia; B-PLL, B-cell Prolymphocytic Leukemia; ABC, Antibodies Bound Per Cell*.

The FC assays were performed on 3 CD19+ malignant B-cell lines expressing CD19 at high (Raji), medium (MEC-1), and low (JVM-2) levels (**Figure S1**). The cells were first incubated with tafasitamab at a wide range of concentrations, washed, and subsequently incubated with a single fluorescently labelled CD19 detection antibody at a constant concentration. In this setup, reduction of the fluorescent signal on tafasitamab-treated cells compared to untreated cells indicates CD19 binding competition (**Figure 2A**). With all 8 clones, fluorescent signal decreased with increasing concentrations of tafasitamab, and was completely abrogated at saturating tafasitamab concentrations (above 10 nM) on all 3 cell lines tested (**Figures 2B**, **S6**). Clone OTI3B10, demonstrated to detect CD19 in IHC, is also marketed for FC on live cells. It appeared not to compete with tafasitamab on Raji cells, but it did not bind to MEC-1 or JVM-2 cells in a FC assessment, which rendered it unsuitable as a FC detection tool (**Figures S8**, **S9**). In summary, the CD19 detection capabilities of all 8 FC antibody clones tested were reduced or diminished on tafasitamab pre-treated cells. Interestingly, therapeutically relevant antibody clones such as FMC63 (CD19 targeting moiety of axi-cel, tisa-cel and liso-cel) and RB4 (loncastuximab tesirine) also competed with tafasitamab and each other (**Figure 2B**, **Figure S10**) (11–14). CD19 affinity characterization revealed that tafasitamab has a higher or similar affinity for CD19 and slower dissociation rate than the other antibodies tested, explaining why tafasitamab was not replaced by the CD19 detection antibodies during FC staining (**Table S7**). Nevertheless, it is important to note that CART cells express multiple chimeric antigen receptors and are thus characterized with avidity, which is not accounted for using the experimental setups of this study. This parameter would be vital to consider from a therapeutic point of view.

As epitope binding competition prevented most screened FC antibodies from detecting CD19, an acidic dissociation protocol was developed, designed to strip tafasitamab from the cell surface prior to staining with a competing CD19 clone (**Figure 2C**). The technique employs incubating the tafasitamab-treated cells using a low pH (pH = 2.1) buffer, leading to dissociation of the antibody from the antigen (15). This method was tested on 4 DLBCL cell lines (U-2932 and SU-DHL-2/4/6) treated with tafasitamab (**Figure 2D**). Acidic dissociation efficiently unmasked CD19 on the surface of all cell lines and allowed for detection of CD19 levels comparable to untreated controls, using a tafasitamab-competing antibody clone.

## Discussion

CD19 is an attractive target for the treatment of B cell malignancies due to its consistent expression at most stages of B cell development. This has led to the development of multiple therapeutic modalities targeting CD19, e.g. naked monoclonal antibodies, antibody-drug conjugates, and chimeric antigen receptor T cells (CART). The availability of different anti-CD19 therapies opens up the possibility to administer them sequentially to patients, which is however connected to concerns relating to potential effects on CD19 expression after treatment with prior CD19-directed therapies.

To address these concerns, the current study provides an overview of commonly used monoclonal antibody tools for CD19 detection post- tafasitamab treatment, some of which may be used in routine clinical practice. Our findings reveal that when attempting to detect CD19 by FC, prior acidic dissociation of tafasitamab from CD19 is critical for accurate measurements of CD19 levels on tafasitamab-treated samples. This acidic dissociation step is especially important, as we have seen that tafasitamab exhibits high affinity to CD19 and a slow dissociation rate compared to the other tested anti-CD19 antibodies. Additionally, performing FC staining with and without acidic dissociation on the same samples can provide information about both CD19 occupancy by tafasitamab and total CD19 levels on the cell surface. When using the IHC antibodies screened in this study, CD19 could be detected independent of tafasitamab treatment, meaning that a wide range of commercially available anti-CD19 antibodies for IHC can be used freely on tafasitamab-treated samples, without the risk of data misinterpretation. Conversely, this also means that those antibodies would not provide information about CD19 occupancy by tafasitamab, and thus potentially the availability of CD19 for binding by subsequent anti-CD19 therapies if the epitopes should overlap.

As IHC was unable to discriminate between Raji and JVM-2 cells in respect to their CD19 levels in our experiments, while FC showed a 5-fold difference in expression, the semi-quantitative nature of IHC was confirmed by our results. However, in the JULIET study testing tisagenlecleucel in DLBCL, similar treatment responses were observed in patients with normal versus low/absent CD19 expression as determined by quantitative immunofluorescence (16), suggesting that accurate determination of CD19 expression levels may not be required when considering eligibility for CART19 therapy. Any positive staining obtained via IHC, regardless of intensity, may be sufficient for subsequent CART19 therapy after prior treatment with other anti-CD19 therapies. This would fit to the hypothesis that CAR T cells are capable of mediating anti-tumor activity against cells with low antigen density (17).

The data outlined in this study is intended to help investigators select appropriate tools for CD19 detection and could serve as a valuable reference in both basic research and clinical practice. Differentiating total from masked antigen on patient biopsies can allow clinical investigators to properly interpret CD19 availability after anti-CD19 therapy, which could be critically important for future treatment strategies.

## About Tafasitamab

Tafasitamab is a humanized Fc-modified cytolytic CD19 targeting monoclonal antibody. In 2010, MorphoSys licensed exclusive worldwide rights to develop and commercialize tafasitamab from Xencor, Inc. Tafasitamab incorporates an XmAb® engineered Fc domain, which mediates B-cell lysis through apoptosis and immune effector mechanism including Antibody-Dependent Cell-Mediated Cytotoxicity (ADCC) and Antibody-Dependent Cellular Phagocytosis (ADCP). In January 2020, MorphoSys and Incyte entered into a collaboration and licensing agreement to further develop and commercialize tafasitamab globally. Following accelerated approval by the U.S. Food and Drug Administration in July 2020, tafasitamab is being co-commercialized by MorphoSys and Incyte in the United States. Conditional/Accelerated approvals were granted by the European Medicines Agency and other regulatory authorities. Incyte has exclusive commercialization rights outside the United States.

XmAb® is a registered trademark of Xencor Inc.

## Data availability statement

The original contributions presented in the study are included in the article/**Supplementary Material**. Further inquiries can be directed to the corresponding author.

## Ethics statement

Ethical approval was not required for the studies on humans in accordance with the local legislation and institutional requirements because only commercially available established cell lines were used. Ethical approval was not required for the studies on animals in accordance with the local legislation and institutional requirements because only commercially available established cell lines were used.

## Author contributions

KI: Conceptualization, Data curation, Formal analysis, Methodology, Supervision, Visualization, Writing - original draft, Writing - review & editing. ME: Data curation, Formal analysis, Methodology, Supervision, Visualization, Writing - original draft, Writing - review & editing. JJ: Data curation, Formal analysis, Methodology, Visualization, Writing - original draft, Writing - review & editing. DB: Writing - review & editing. MP-K: Writing - review & editing. RB: Conceptualization, Writing - review & editing. DA: Writing - review & editing. CH: Conceptualization, Supervision, Writing - review & editing.
