# Supplementary material for: Preclinical study of CD19 detection methods post tafasitamab treatment
Source: Front Immunol. 2023 Oct 20;14:1274556. doi: 10.3389/fimmu.2023.1274556 (PMC10622958; doi:10.3389/fimmu.2023.1274556)
Supplement: Supplementary file 1 [file DataSheet_1.docx]

**Supplementary Materials and Methods**

**Supplementary Table 1 (S1)**

List of CD19 detection antibodies for immunohistochemistry

| **Clone** | **Supplier** | **Catalogue number** | **Regulatory Status** | **Conjugate** | **CD19 epitope** | **Reported application by supplier (detection)** | **Dilution** | **Buffer** | **Note** |
| --- | --- | --- | --- | --- | --- | --- | --- | --- | --- |
| BT51E | Leica | NCL-L-CD19-163 | IVD | N/A | ICD | IHC | 1:200 | Citrate | Std* |
| LE-CD19 | Bio-Rad | MCA2454 | RUO | N/A | ICD | FC (permeabilized), IHC /frozen), IHC (paraffin), ELISA, IP, WB | 1:2000 | EDTA | Std |
| D4V4B | Cell Signalling Technology | 90176 | RUO | N/A | ICD | WB, IP, IHC (paraffin), IHC Leica Bond, FC (permeabilized) | 1:200 | Citrate | Std |
| OTI3B10 | Origene | TA506236 | RUO | N/A | ECD | FC, IF, IHC, WB | 1:150 | Citrate | Std |
| 109 | Thermo Fisher Scientific | MA5-29094 | RUO | N/A | ECD | IHC (paraffin) | 1:200 | EDTA | Std |
| ZR212 | GeneTex | GTX01616 | RUO | N/A | ECD | IHC (paraffin) | 1:200 | EDTA | Std |
| 3117 | Abcam | ab270715 | RUO | N/A | ECD | IHC (paraffin), protein array | 1:1000 | EDTA | Std |
| 25 | SinoBiological | 11880-MM25 | RUO | N/A | ECD | IHC (paraffin) | N/A | N/A | ND** |
| 3116 | NSJ Bioreagents | V8224 | RUO | N/A | ECD | ELISA, FC, IHC (paraffin) | N/A | N/A | ND |
| FMC63 | Novus Biologicals | NBP2-52716 | RUO | N/A | ECD | ELISA, FC, ICC/IF, IHC, IHC (paraffin), CyTOF | N/A | N/A | ND |
| HD37 | Absolute antibody | Ab00214-23.0 | RUO | N/A | ECD | IP, FC, IF, IHC | N/A | N/A | ND |
| Polyclonal | Thermo Fisher Scientific | bs-0079R | RUO | N/A | N/A | WB, IHC (paraffin), IHC (frozen), ICC/IF, FC, ELISA | N/A | N/A | ND |

*Std – standard IHC treatment

**ND - No suitable staining conditions were identified for this antibody. No specific signal was observed in positive control tissue (=human lymph node) using the specific IHC methodology employed in this manuscript

Abbreviations: ECD – extracellular domain; ICD – intracellular domain; IP – Immunoprecipitation; ChIP - Chromatin Immunoprecipitation; C&R – Cut and Run; C&T – Cut and Tag; DB – Dot Blot, IF – immunofluorescence; ELISA – Enzyme Linked Immunosorbent Assay; ICC – immunocytochemistry; CyTOF – Cytometry by Time of Flight; EDTA – Ethylenediaminetetraacetic Acid; RUO = research use only; IVD = in vitro diagnostic

**Supplementary Table 2 (S2)**

List of immunohistochemistry tool antibodies

| **Clone** | **Supplier** | **Catalogue number** | **Regulatory status** | **Target** | **Species/isotype** | **Conjugate** | **Dilution** | **Buffer** | **Note** |
| --- | --- | --- | --- | --- | --- | --- | --- | --- | --- |
| EPR4421 | Abcam | Ab109489 | RUO | Human IgG | Rabbit IgG | N/A | 1:1000 | EDTA | Incubation of antibody for 1 h at RT only. Detection via SignalStain® Boost IHC Detection Reagent  (cat.# 8114) |
| I17-T | Abcam | Ab180635 | RUO | Human IgG | Rabbit IgG | N/A | 1:1000 | EDTA | Incubation of antibody for 1 h at RT only. Detection via SignalStain® Boost IHC Detection Reagent  (cat.# 8114) |
| Polyclonal | Jackson ImmunoResearch | 115-067-003 | RUO | Mouse IgG (H+L) | Goat Fab | Biotin-SP^TM^ | 1:400 | N/A | Detection antibody for standard IHC treatment |
| Polyclonal | Jackson ImmunoResearch | 111-067-003 | RUO | Rabbit IgG (H+L) | Goat Fab | Biotin-SP^TM^ | 1:400 | N/A | Detection antibody for standard IHC treatment |
| N/A | Cell Signaling | 8114 | RUO | Rabbit IgG | Goat | HRP-Polymer | Ready to use / undiluted | N/A | Used as detection antibody for clone EPR4421 and I17-T |
| DA1E | Cell Signalling | 3900 | RUO | N/A (isotype control) | Rabbit IgG | N/A | Same as respective specific antibody | Same as respective specific antibody | Used as an isotype control for clones 109, ZR212 and D4V4B |
| MOPC-21 | BioLegend | 400166 | RUO | N/A (isotype control) | Mouse IgG1 | N/A | Same as respective specific antibody | Same as respective specific antibody | Used as an isotype control for clone OTI3B10 and LE-CD19 |
| E7Q5L | Cell Signalling | 53484 | RUO | N/A (isotype control) | Mouse IgG2b | N/A | Same as respective specific antibody | Same as respective specific antibody | Used as an isotype control for clones 3117 and BT51E |

Abbreviations: H+L – Heavy + Light; Biotin-SP – Biotin Long-Spacer; HRP – Horseradish Peroxidase;

**Supplementary Table 3 (S3)**

List of CD19 detection antibodies for flow cytometry

| **Clone** | **Supplier** | **Cat Number** | **Regulatory status** | **Conjugate** | **Reported application by supplier(detection)** | **Note** |
| --- | --- | --- | --- | --- | --- | --- |
| HIB19 | BioLegend | 302208 | RUO | Phycoerythrin (PE) | FC, IHC of acetone-fixed frozen tissue | N/A |
| 4G7 | BD Bioscience | 345777 | CE_IVD | Phycoerythrin (PE) | FC | N/A |
| FMC63 | Merck Millipore | MAB1794H | RUO | Phycoerythrin (PE) | FC | N/A |
| SJ25C1 | BD Bioscience | 345789 | CE_IVD | Phycoerythrin (PE) | FC | N/A |
| J3-119 | Beckman Coulter | A07769 | CE, Japan IVD | Phycoerythrin (PE) | FC | N/A |
| LT19 | Miltenyi Biotec | 130-113-169 | CE_IVD | Phycoerythrin (PE) | FC | N/A |
| REA675 | Miltenyi Biotec | 130-113-646 | RUO | Phycoerythrin (PE) | FC, IHC, MICS | N/A |
| OTI3B10 | Origene | TA506236HM | N/A | Phycoerythrin (PE) | FC, IF, IHC, WB | N/A |
| HD37 | Absolute antibody | Ab00214-23.0 | IP, FC, IF, IHC | N/A | IP, FC, IF, IHC | Detected using a secondary antibody cat# 111-116-144 (see Table S4) |

Abbreviations:

CE_IVD – Conformite Europeenne – In Vitro Diagnostics; MICS – MACSima Imagine Cyclic Staining; FC – Flow Cytometry; WB – Western Blot

**Supplementary Table 4 (S4)**

List of flow cytometry tool antibodies

| **Clone** | **Supplier** | **Catalogue number** | **Regulatory status** | **Target** | **Species/isotype** | **Conjugate** | **Dilution** | **Note** |
| --- | --- | --- | --- | --- | --- | --- | --- | --- |
| MOPC-21 | BioLegend | 400112 | RUO | N/A (isotype control) | mIgG1 | PE | same as respective specific antibody | Used as an isotype control for clones J3-119, 4G7 SJ25C1, OTI3B10, HIB19, LT19, REA675 |
| MOPC-173 | BioLegend | 400214 | RUO | N/A (isotype control) | mIgG2a | PE | same as respective specific antibody | Used as an isotype control for clone FMC63 |
| DA1E | Cell Signalling | 3900 | RUO | N/A | Rabbit IgG | N/A | same as respective specific antibody | Used as an isotype for clone HD37 |
| Polyclonal | Jackson ImmunoResearch | 111-116-144 | In vitro RUO | Rabbit IgG (H+L) | Goat F(ab´)_2_ | PE | 1:100 | Used to detect clone HD37 |
| Polyclonal | Jackson ImmunoResearch | 109-096-088 | In vitro RUO | Human IgG (H+L) | Goat F(ab´)_2_ | FITC | 1:100 | Used to detect human IgG/tafasitamab |
| Polyclonal | Jackson ImmunoResearch | 109-116-098 | In vitro RUO | Human IgG, Fcy specific | Goat F(ab´)_2_ | PE |  | Used to detect human IgG |
| Polyclonal | Jackson ImmunoResearch | 109-136-170 | In vitro RUO | Human IgG, Fcy specific | Goat F(ab´)_2_ | APC |  | Used to detect human IgG |

AbbPE – phycoerythrin; FITC – Fluorescein Isothiocyanate; APC – Allophycocyanin;

**Supplementary Table 5 (S5)**

List of anti-CD19 antibodies used for affinity measurements

| **Clone** | **Supplier** | **Cat Number** | **Status** | **Conjugate** | **Species and isotype** | **Reported application** |
| --- | --- | --- | --- | --- | --- | --- |
| Tafasitamab | MorphoSys AG | N/A | N/A | N/A | hIgG1 | N/A |
| J3-119 | Beckman Coulter | IM1313 | ASR | N/A | mIgG1 | FC |
| 4G7 | Thermo Fisher Scientific | MA1-10126 | RUO | N/A | mIgG1 | FC, ICC/IF |
| SJ25C1 | BioLegend | 363001/363002 | RUO | N/A | mIgG1 | FC, IHC (frozen) |
| LT19 | Miltenyi Biotec | 130-108-029 | RUO | N/A | mIgG1 | FC |
| HIB19 | BioLegend | 302267/302268 | RUO | N/A | mIgG1 | FC, CyTOF |
| OTI3B10 | Origene | TA506236 | N/A | N/A | mIgG1 | FC, IF, IHC, WB |
| FMC63 | Novus Biologicals | NBP2-52716 | RUO | N/A | mIgG2 | ELISA, FC, ICC/IF, IHC, IHC (paraffin), CyTOF |
| REA675 | Miltenyi Biotec | 130-122-301 | RUO | N/A | Recombinant human IgG1 | FC |
| RB4 | Recombinantly expressed in house | N/A | N/A | N/A | hIgG1 | N/A |
| HD37 | Absolute antibody | Ab00214-23.0 | RUO | N/A | chimeric rabbit IgGk, mIgG1 variable domains | IP, FC, IF, IHC |

Abbreviations: ASR – Analyte Specific Reagent;

**Supplementary Table 6 (S6)**

List of buffers used for affinity measurements

| **Buffer** | **Composition** | **pH** |
| --- | --- | --- |
| HBS-EP+ | 10 mM HEPES, 150 mM NaCl (sodium chloride), 3 mM EDTA, 0.05% v/v surfactant P20 | 7.4 |
| PBST+B | 8.06 mM Na_2_HPO_4_-7H_2_O (sodium phosphate dibasic), 2.67 mM KCl (potassium chloride), 1.47 mM KH_2_PO_4_ (potassium phosphate monobasic), 137.93 mM NaCl,  1% w/v bovine serum albumin, 0.05% v/v polysorbate 20 | ~7.1 (7.0-7.4) |
| Regeneration solution 1 | 10 mM Glycine/HCl | 1.7 |
| Regeneration solution 2 | 10 mM Glycine/HCl | 1.5 |

**Affinity measurements**

**Antigen**

Recombinant human CD19 was produced and purified as monomeric protein with a molecular weight of 46.1 kDa. The construct consisted of the extracellular domain of CD19 20-277, a chicken lysozyme-tag and an avi-tag.^1^ It was expressed in HKB11 cells, stably transfected with a proprietary vector encoding for the protein and affinity-purified using a column coupled with a lysozyme-specific antibody (produced at MorphoSys AG)^[[1]](#footnote-1)^. In analytical high-performance size-exclusion chromatography (HP-SEC), the protein was found to contain >97% monomeric main peak.

**Kinetic characterization of the interaction between human CD19 and CD19-specific antibodies by surface plasmon resonance (SPR)**

CD19-targeting antibodies (Table S5) were characterized in terms of kinetics and affinity to their target (human CD19, monomer). All samples were analyzed in an IgG capture setup, with high surface capacities of capture ligands covalently immobilized on CM5 SPR sensors (Cytiva; 29149603). Murine antibodies were captured by anti-mouse Fc (Cytiva, # BR100838) and human and rabbit IgGs were captured by MabSelect SuRe ligand (Cytiva, # 28-4018-60).

IgG samples were diluted to a concentration in the range of 7 to 100 nM and injected for a duration which allowed to achieve capture levels of ~ 60 RU (up to ~ 180 RU for clone 4G7). Human CD19 protein was diluted in assay running buffer to the highest applied assay concentration (adapted to the observed affinities of the IgG samples; 80 to 600 nM) from which additional analyte concentrations were prepared following two- or three-fold serial dilutions. Association and dissociation were recorded at a flow rate of 30 µL/min. The duration of association and dissociation were adapted to the observed kinetics of the IgG samples interacting with CD19 to allow for sufficient curvature during binding and significant dissociation. Association times between 120 and 420 s were applied, and dissociation was monitored for 300 – 1200 s.

k_on_, k_off_, K_D_ values and activity are presented as averages of n independent experiments ± standard deviation (Table S7). The activity was calculated as experimentally observed or extrapolated saturation level Rmax, divided by the expected theoretical saturation level (see formulas below). The theoretical saturation level was calculated based on the capture level (CL), molecular weights (MW) of CD19 and antibody, and an expected interaction of 2 molecules of (monomeric) CD19 per captured IgG.

Rmax (theor.) = 2 * MW(CD19) / MW(IgG) * CL

Activity = Rmax (experimental) / Rmax (theor.)

The recorded sensorgrams of the different CD19 concentrations for each antibody were reference- and blank-subtracted, and fitted to a 1:1 kinetic model, with parameters k_on_, k_off_, Rmax (saturation) fitted globally. RI (refractive index) was set to constant and zero, except for the evaluation of clone 4G7, where allowing the RI variable to be fitted allowed a more robust approximation of the monovalent fit to this interaction (which deviated from monovalent kinetics). For other samples, sensorgrams were not included in the evaluation if they either (a) deviated from the expected monovalent binding (typically occurred at the high end of the CD19 dilution series), or (b) did not show significant binding and thus did not contribute to the description of the kinetics (typically at the lowest CD19 concentration(s)).

**Kinetic characterization of the interaction between human CD19 and anti-CD19 antibody clone OTI3B10 by biolayer interferometry (BLI)**

The interaction of clone OTI3B10 was initially investigated by SPR. Due to slow association and dissociation rate constants, and limited association times, the interaction was also characterized by BLI using extended association times. IgG capture setup was used for BLI analyses. OTI3B10 was diluted to 30 nM and captured on anti-mouse Fc (AMC) BLI sensors (Sartorius, 18-5089) until a capture level of ~ 0.8 nm was achieved. Human CD19 was used as an analyte in solution at concentrations ranging from 1000 to 31.25 nM (2-fold dilution series). Association and dissociation times were tested and increased stepwise starting from 15 min association and 20 min dissociation to a maximum of 30 min association and 40 min dissociation. At the end of the interaction, captured IgG (together with bound CD19) was removed from the sensor by two 40 s regeneration cycles with Glycine / HCl at pH 1.7.

After blank subtraction against the analyte at a concentration of zero, the recorded sensorgrams were fitted to a monovalent 1:1 binding model.

**Supplementary Results**

**FC competition of anti-CD19 antibody clones, therapeutically relevant in DLBCL**

Using FC, we tested whether 1) clones FMC63 (CD19 targeting moieties of axicabtagen-ciloleucel, lisocabtagene maraleucel and tisagenlecleucel) and RB4 (CD19-targeting moiety of loncastuximab tesirine) compete with tafasitamab (Fig. S10A, B, Fig 2B); 2) clone FMC63 competes with RB4 (Fig. S10C); 3) tafasitamab competes with RB4 (Fig. S10D). FMC63 and RB4 were both demonstrated to compete with tafasitamab as the fluorescent signals from both antibodies were suppresed at tafasitamab concentrations over 10 nM (Fig. S10A, B). FMC63 was also shown to compete with RB4 as its fluorescent signal was reduced with increasing concentrations of RB4 (Fig. S10C). In this case, the FMC63 signal was not completely suppressed at saturating concentrations of RB4, likely due to the higher affinity of FMC63 for CD19 (Table S7). Similarly, tafasitamab competed with RB4 but saturating concentrations of RB4 did not completely block tafasitamab binding to CD19.

**CD19 detection using antibody clone OTI3B10**

CD19 antibody clone OTI3B10, reported to recognize a linear epitope, was tested by IHC and FC on live cells as per manufactures recommendation (Table S1, S3).^2^ OTI3B10 did not compete with tafasitamab on Raji cells by FC. However, we could not test competition with tafasitamab due to lack of OTI3B10 binding to MEC-1 and JVM-2 cells (Fig. S8, Fig. S9). Interestingly, clone OTI3B10 was able to detect CD19 on JVM-2 cells using IHC, highlighting an apparent differential recognition pattern between the native and the denatured CD19 protein by this antibody (Fig. 1B). Affinity measurements indicated that OTI3B10 binds CD19 with considerably lower affinity (K_D_ = 19.4 nM) than the other anti-CD19 clones (Table S7). Since antibodies of lower affinity display stronger dependency on the density of their cognate antigens on the cell surface, the observed low affinity of OTI3B10 for CD19 may explain its behaviour as a CD19 detection antibody for FC.^3^

**Affinity measurements**

Most samples largely followed the expected 1:1 binding kinetics (with some deviation at higher CD19 concentrations) and could consequently be well described by the corresponding monovalent kinetic fit model (Table S7, S8).

Clone 4G7 was inactive, i.e., only a portion (approx. 16%) appeared to be binding competent for CD19, which was considered either a characteristic of the antibody or of the specific investigated Lot, used in the experiment (Table S7, S8). Due to the low antibody activity and deviation from monovalent kinetics, the quantitative results presented in Table S7 should be regarded as an order of magnitude or an approximation rather than results with the same descriptive value as the results generated for the other clones.

Clone OTI3B10 showed unusually slow association and dissociation rates. Due to the longer association times which could be realized by BLI compared to SPR, the final characterization of OTI3B10 was carried out by Octet / BLI which essentially confirmed the observations made using SPR, but with better data quality (better described association with more curvature) (Table S8).

**Supplementary figures**

**Supplementary Figure 1 (S1).** Quantification of CD19 expression on Raji, MEC-1 and JVM-2 cells. CD19 molecules per cell were quantified using the BD Quantibrite^TM^ system. Representative example, measurement was performed on a regular basis. ABC – Antibodies Bound Per Cell

**Supplementary Figure 2 (S2).**  Flow cytometric analysis of Raji, MEC-1 and JVM-2 cells prior to fixation. Before cell pellet preparation, cells were treated with 50 nM tafasitamab or left untreated and analyzed by FC. To confirm CD19 was completely blocked by tafasitamab, the tafasitamab-competing clone HIB19 PE was used for detection. Tafasitamab bound to CD19 on the cell surface was detected using an anti-human IgG FITC. MFI – Median Fluorescence Intensity

**Supplementary Figure 3 (S3).** FC analysis of surface IgG expression on Raji and JVM-2 cells. To confirm lack of surface IgG, which could interfere with tafasitamab detection in immunohistochemistry, the cells were stained using two different polyclonal antibodies against human IgG. Unstained cells and cells incubated with 50 nM tafasitamab + anti-human IgG were used as controls.


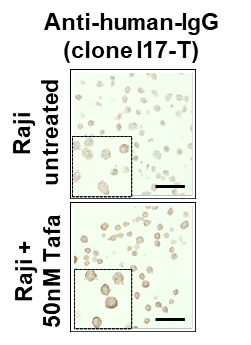


scale bar = 50µm)

**Supplementary Figure 4 (S4).** Staining of untreated and tafasitamab-incubated Raji cell pellets using an anti-human IgG antibody. Clone I17-T was used to confirm that tafasitamab is still present in the samples and not lost during pellet processing. Sample acquisition and analysis specifics were as described in the legend of Fig. 1B.

1. **B.**


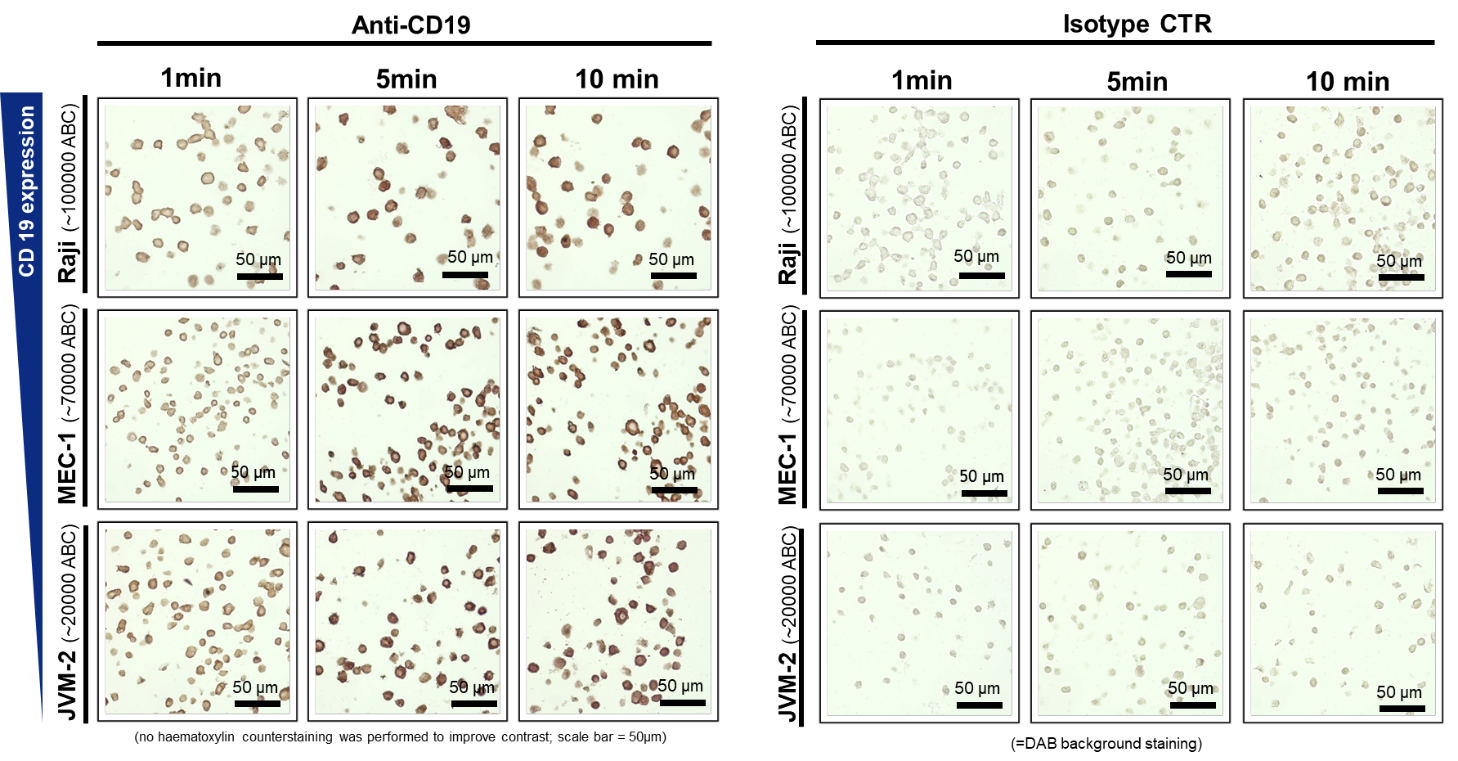


**Supplementary Figure 5 (S5)** **A.** DAB staining optimization. Three different staining times (1min, 5min and 10min) were tested using DAB substrate for clone LE-CD19: maximum staining intensity was reached after 5 min. **B.** Background staining for the respective isotype control antibody MOPC-21 remained unchanged at 1min, 5min and 10min. Representative example is shown. The test was performed for all other IHC detection antibodies with cell lines Raji and JVM-2 and for clone 109 with all three cell lines. Sample acquisition and analysis specifics were as described in the legend of Fig. 1B. DAB – 3,3´-diaminobenzidine

**Supplementary Figure 6 (S6)**. Titration of tafasitamab on Raji, MEC-1 and JVM-2 cells by FC. Tafasitamab-AF488 was titrated down starting from 50nm (5-fold dilution). AF488 – Alexa Fluor 488

**Supplementary Figure 7 (S7).** FC analysis of CD19 clones binding to Raji cells. Antibodies were titrated down starting from 50nm (clones 4G7, SJ25C1) or 150 nM (clones FMC63, HIB19, RB4 and HD37), (3-fold dilution). PE-conjugates of clones 4G7, SJ25C1, FMC63 and HIB19 were used. Clone RB4 bearing an mScarlet fluorescent tag was produced in house, while HD37 was detected using a PE-conjugated secondary anti-rabbit IgG antibody.

**Supplementary Figure 8 (S8).** Flow cytometric analyses of OTI3B10 binding to Raji, MEC-1 and JVM-2 cells. Antibody clone OTI3B10 was titrated down starting from 333 nM (3-fold dilution). Clone HIB19 was used as a positive control. PE – Phycoerythrin; MFI – Median Fluorescence Intensity; Iso Ctrl – Isotype Control

**Supplementary Figure 9 (S9).** Flow cytometry competition of OTI3B10 with tafasitamab. Cells were pre-incubated with different concentrations of tafasitamab (I), washed and incubated with 50 nM OTI3B10-PE (II). Reduction/loss of the PE fluorescent signal was considered an indicator of competition. n=3 independent experiments; error bars indicate SD; PE – Phycoerythrin; MFI – Median Fluorescence Intensity; Iso Ctrl – Isotype Control; SD – Standard Deviation.

I + II antibody

II antibody alone

**Supplementary Figure 10 (S10).** FC competition assay of tafa with other therapeutically relevant anti-CD19 antibodies: FMC63 – CD19 targeting moiety of axicabtagen-ciloleucel, lisocabtagene maraleucel and tisagenlecleucel; RB4 – CD19 targeting moiety of loncastuximab tesirine.^4-7^ Cells were incubated with the first antibody at different concentrations (I), washed and incubated with 50 nM of a second, fluorescently labelled anti-CD19 antibody (II). A commercial PE-conjugate of FMC63, in house produced RB4 bearing a fluorescent mScarlet tag and in house produced AF488-conjugate of tafasitamab were used in step II. n=3 independent experiments; error bars indicate SD

**Supplementary Table 7 (S7).** **Numerical results of kinetic characterization (by SPR or BLI).** Results are presented as an average ± SD of n experiments.

| **Sample name** | **n** | **k_on_  [1/Ms]** | **k_off_  [1/s]** | **K_D_ [nM]** | **Activity [%]** |
| --- | --- | --- | --- | --- | --- |
| tafasitamab | 3 | 1.2 ± 0.2 E+5 | 1.1 ± 0.2 E-4 | 1.0 ± 0.2 | 111 ± 2 |
| clone J3-119 | 4 | 3.1 ± 0.8 E+5 | 2.9 ± 0.3 E-4 | 1.0 ± 0.5 | 71 ± 7 |
| clone 4G7 * | 5 | ( 5.6 ± 2.4 E+4 ) | ( 5.7 ± 1.1 E-4 ) | ( 12.0 ± 5.7 ) | 16 ± 3 |
| clone SJ25C1 | 3 | 1.9 ± 0.9 E+5 | 1.9 ± 0.1 E-3 | 11.4 ± 4.3 | 102 ± 15 |
| clone LT19 | 3 | 1.5 ± 0.3 E+5 | 6.4 ± 0.1 E-4 | 4.5 ± 1.1 | 108 ± 14 |
| clone HIB19 | 3 | 1.8 ± 0.8 E+5 | 2.2 ± 0.1 E-3 | 13.6 ± 4.8 | 101 ± 2 |
| clone OTI3B10 ** | 5 | 3.1 ± 0.8 E+3 | 5.9 ± 2.4 E-5 | 19.4 ± 7.8 | 67 ± 4 |
| clone FMC63 | 3 | 1.6 ± 0.5 E+5 | 5.0 ± 0.5 E-4 | 3.4 ± 1.2 | 78 ± 6 |
| clone REA675 | 3 | 3.3 ± 0.2 E+5 | 3.9 ± 0.9 E-3 | 11.9 ± 3.2 | 110 ± 14 |
| clone RB4  (INN loncastuximab) | 3 | 3.6 ± 0.0 E+5 | 2.0 ± 0.0 E-3 | 5.4 ± 0.1 | 115 ± 5 |
| clone HD37 | 3 | 6.1 ± 0.3 E+5 | 2.5 ± 0.1 E-3 | 4.1 ± 0.3 | 115 ± 1 |

*Low activity of IgG, deviation from monovalent kinetics

**Slow k_on_, slow k_off_. Reported data was generated using BLI. SPR experiments gave similar results, though with reduced data quality (due to less curvature during association phase).

Supplementary Table 8 (S8) Representative sensorgram overlays (SPR or BLI), in blue. The applied monovalent fit is shown in black (SPR) or red (BLI).

|  | MOR208 (INN tafasitamab)  included conc.: 2.5 – 200 nM  association: 300 s  (max.) dissociation: 1200 s  k_a_ = 1.3 E+5 (Ms)^-1^  k_d_ = 9.9 E-5 (s)^-1^  K_D_ = 0.7 nM  activity of IgG ligand = 109 % |
| --- | --- |
|  | Clone J3-119  included conc.: 2.5 - 66.7 nM  association: 300 s  (max.) dissociation: 1200 s  k_a_ = 3.0 E+5 (Ms)^-1^  k_d_ = 2.6 E-4 (s)^-1^  K_D_ = 0.9 nM  activity of IgG ligand = 62 % |
|  | Clone 4G7  included conc.: 12.5 - 400 nM  association: 420 s  (max.) dissociation: 1200 s  k_a_ = 2.7 E+4 (Ms)^-1^  k_d_ = 5.6 E-4 (s)^-1^  K_D_ = 21 nM  activity of IgG ligand = 17 %  **low activity, heterogeneous binding, RI fitted** |
|  | Clone SJ25C1  included conc.: 6.25 - 200 nM  association: 300 s  (max.) dissociation: 1200 s  k_a_ = 2.9 E+5 (Ms)^-1^  k_d_ = 2.0 E-3 (s)^-1^  K_D_ = 7.1 nM  activity of IgG ligand = 96 % |
|  | Clone LT19  included conc.: 2.5 - 200 nM  association: 300 s  (max.) dissociation: 1200 s  k_a_ = 1.6 E+5 (Ms)^-1^  k_d_ = 6.4 E-4 (s)^-1^  K_D_ = 4.0 nM  activity of IgG ligand = 102 % |
|  | Clone HIB19  included conc.: 6.25 - 100 nM  association: 300 s  (max.) dissociation: 1200 s  k_a_ = 2.7 E+5 (Ms)^-1^  k_d_ = 2.2 E-3 (s)^-1^  K_D_ = 8.4 nM  activity of IgG ligand = 100 % |
| 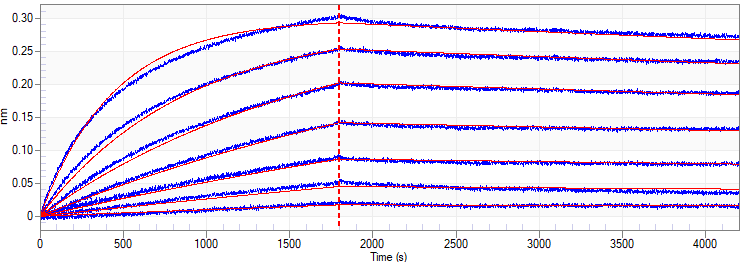 | Clone OTI3B10 (by BLI)  included conc.: 15.6 - 1000 nM  association: 1800 s  (max.) dissociation: 2400 s  k_a_ = 2.2 E+3 (Ms)^-1^  k_d_ = 3.9 E-5 (s)^-1^  K_D_ = 17 nM  activity of IgG ligand = 71 % |
|  | Clone FMC63  included conc.: 6.25 - 100 nM  association: 300 s  (max.) dissociation: 1200 s  k_a_ = 1.7 E+5 (Ms)^-1^  k_d_ = 5.6 E-4 (s)^-1^  K_D_ = 3.2 nM  activity of IgG ligand = 85 % |
|  | Clone REA675  included conc.: 3.13 - 100 nM  association: 240 s  (max.) dissociation: 600 s  k_a_ = 3.2 E+5 (Ms)^-1^  k_d_ = 5.0 E-3 (s)^-1^  K_D_ = 16 nM  activity of IgG ligand = 94 % |
|  | Clone RB4 (INN ioncastuximab)  included conc.: nM  association: s  (max.) dissociation: s  k_a_ = 3.6 E+5 (Ms)^-1^  k_d_ = 2.0 E-3 (s)^-1^  K_D_ = 5.5 nM  activity of IgG ligand = 112 % |
|  | HD37  included conc.: 1.25 – 80 nM  association: 120 s  dissociation: 300 s  k_a_ = 6.0 E+5 (Ms)^-1^  k_d_ = 2.5 E-3 (s)^-1^  K_D_ = 4.2 nM  activity of IgG ligand = 114 % |

All assay conditions and results in the right column refer to the specific replicate experiment presented in the left part of the table, i.e. represent one of the different replicate experiments contributing to the average values presented in table S7.

1. Haertle S, Jaeger S, Daubert D. Use of lysozyme as a tag. *US201261589408P;EP20120152095;WO2013EP51181* (2013) CA CA2860916 (A1)
2. Klesmith JR, Wu L, Lobb RR, Rennert PD, Hackel BJ. Fine Epitope Mapping of the CD19 Extracellular Domain Promotes Design. *Biochemistry*. /2019) 58(48):4869-4881. doi: 10.1021/acs.biochem.9b00808.
3. Hadzhieva M, Pashov AD, Kaveri S, Lacroix-Desmazes S, Mouquet H, Dimitrov JD. Impact of Antigen Density on the Binding Mechanism of IgG Antibodies. *Sci Rep* (2017) 7(1):3767. doi: 10.1038/s41598-017-03942-z.
4. Assessment report Yescarta. *European Medicines Agency* (2018) [h-4480-par-EN (europa.eu)](https://www.ema.europa.eu/en/documents/assessment-report/yescarta-epar-public-assessment-report_en.pdf)
5. Assessment report Kymriah. *European Medicines Agency.* (2018) [ema.europa.eu/en/documents/assessment-report/kymriah-epar-public-assessment-report_en.pdf](https://www.ema.europa.eu/en/documents/assessment-report/kymriah-epar-public-assessment-report_en.pdf)
6. Assessment report Tecartus. *European Medicines Agency.* (2020) [Tecartus - autologous anti-CD19-transduced CD3+ cells (europa.eu)](https://www.ema.europa.eu/en/documents/assessment-report/tecartus-epar-public-assessment-report_en.pdf)
7. Zammarchi F, Corbett S, Adams L, Tyrer PC, Kiakos K, Janghra N, et al. ADCT-402, a PBD dimer-containing antibody drug conjugate targeting CD19-expressing malignancies. *Blood* (2018) 131(10):1094-1105. doi: 10.1182/blood-2017-10-813493.

1. [↑](#footnote-ref-1)
